# Development and Validation of the Cheek Smoothness Scale: A Photonumeric Assessment Tool for Cheek Skin Texture

**DOI:** 10.1111/jocd.70806

**Published:** 2026-03-27

**Authors:** Hye Sung Han, Beom Joon Kim

**Affiliations:** ^1^ Department of Dermatology Institute of Clinical Medicine, Chung‐Ang University Gwangmyeong Hospital, Chung‐Ang University College of Medicine Gwangmyeong‐si Gyeonggi‐do Republic of Korea; ^2^ Department of Dermatology Chung‐Ang University Hospital, Chung‐Ang University College of Medicine Seoul Republic of Korea

**Keywords:** cheek skin, photonumeric scale, scale validation, skin texture, surface roughness

## Abstract

**Background:**

Changes in cheek skin texture and surface smoothness are prominent features of facial aging and increasingly targeted by modern aesthetic interventions. However, despite rising demand for minimally invasive aesthetic procedures to improve cheek skin quality, validated site‐specific photonumeric assessment tools remain limited.

**Aims:**

To develop and validate a novel photonumeric assessment tool, the Cheek Smoothness Scale (CSS), for evaluating cheek skin smoothness and surface texture.

**Methods:**

The CSS was developed as a five‐grade photonumeric scale using standardized clinical photographs. Inter‐ and intra‐rater reliability were assessed using Fleiss' and Cohen's kappa statistics based on single‐angle (45°) and multi‐angle image assessments by five board‐certified dermatologists. Construct validity was further examined by objective skin surface roughness measurements (Ra values) obtained using the PRIMOS CR three‐dimensional imaging system.

**Results:**

The CSS demonstrated excellent inter‐ and intra‐rater reliability, with kappa values consistently in the good‐to‐very good range. Complete agreement between single‐ and multi‐angle assessments supported the use of the 45° oblique view as a representative angle for evaluating cheek skin texture. CSS grades also showed a progressive increase in PRIMOS‐derived Ra values, indicating strong concordance between visually assessed skin smoothness and objectively measured surface roughness.

**Conclusions:**

The CSS is a reliable, reproducible, and clinically meaningful photonumeric scale for assessing cheek skin smoothness and surface texture. By integrating visual assessment with objective validation of surface smoothness, the CSS provides a valuable outcome measure for clinical research and real‐world aesthetic practice.

## 1 Introduction

Alterations in skin texture and surface smoothness are among the most visible features of facial aging and strongly influence overall facial appearance and perceived age. Beyond volumetric changes and deep wrinkles, age‐related skin surface deterioration—manifesting as roughness, unevenness, and loss of smoothness—has emerged as a key determinant of skin quality [1, 2, 3]. These changes are particularly pronounced in the cheek region, which occupies a large central facial area and is continuously exposed to intrinsic aging processes and extrinsic environmental factors, including ultraviolet radiation.

In parallel with these observations, aesthetic practice over the past two decades has seen a marked shift toward minimally invasive interventions aimed at improving skin quality. Treatments such as botulinum toxin type A injections [4, 5, 6], injectable skin boosters [7, 8], and minimally invasive energy devices [9, 10, 11] are increasingly used to target fine textural irregularities and surface smoothness of the skin. However, accurate evaluation of treatment outcomes in this region remains challenging. Visual assessment of cheek skin texture is inherently difficult because age‐related changes are often subtle, diffuse, and highly dependent on lighting conditions and viewing angle [12, 13]. Although a skin roughness scale with defined grade descriptors has been previously described [14], its validation included limited representation of Asian populations and did not incorporate adjunctive objective or visual imaging modalities. Consequently, further validation remains necessary to support its applicability in contemporary clinical research settings.

In this context, we developed the Cheek Smoothness Scale (CSS), a novel five‐grade photonumeric scale for assessing cheek skin texture and surface smoothness using standardized clinical photography with adjunctive objective imaging support. The aim of the present study was to evaluate the reliability of the CSS and to establish its suitability as a practical outcome measure for assessing cheek skin quality in both clinical research and real‐world aesthetic practice.

## 2 Materials and Methods

### 2.1 Study Design and Participants

This study developed and validated the CSS, a five‐grade photonumeric scale ranging from 0 (none) to 4 (very severe).

Overall, 50 adult men and women aged ≥ 19 years were enrolled, with at least nine participants per CSS grade (0–4). Eligible participants were healthy individuals without active facial skin diseases that could interfere with the visual assessment of cheek skin texture.

This study was reviewed and approved by an Institutional Review Board prior to initiation. All participants provided written informed consent, including explicit consent for standardized facial photography and publication of anonymized images in scientific journals. To protect participant privacy, identifying metadata were removed from all image files prior to analysis. Images were stored on secure, access‐restricted servers, and photographs were framed and cropped to minimize identifiable facial features while preserving the cheek region required for grading.

### 2.2 Image Collection and Standardization

Facial photographs were taken using a Canon EOS 850D camera (Japan) under consistent lighting and environmental conditions, with the following settings: resolution 6000 × 3368 pixels, shutter speed 1/125 s, aperture f/11, and ISO 1600. All photographs were captured by the same trained photographer. Participants were seated upright, instructed to look straight ahead, and asked to pull their hair back to fully expose the forehead and cheeks. The facial midline was aligned with the vertical center grid line, and the region from the infraorbital area to the chin tip was included in the frame. Photographs were taken in this order: frontal, right oblique (45°), right lateral (90°), left oblique (45°), and left lateral (90°).

Prior to photography, participants were instructed to remove makeup and cleanse the faces using a gentle cleanser. Images were obtained after an acclimation period of at least 20 min in a temperature‐controlled room to minimize transient erythema or surface hydration changes. Lighting conditions were standardized using fixed studio lighting with consistent positioning and intensity throughout all sessions. The camera‐to‐subject distance and head positioning were kept constant using floor markers and a fixed seating setup.

The cheek region of interest (ROI) was defined anatomically as the area extending from the infraorbital rim to the nasolabial fold and laterally toward the zygomatic prominence. Images were framed consistently to include the full cheek region required for grading. Post‐processing was limited to standardized cropping and file labeling. No retouching, smoothing, contrast enhancement, or local image manipulation was performed.

Photographs were obtained from 50 participants, with bilateral cheek images collected, yielding 100 images. After excluding 7 images due to acquisition errors, 93 images were included for photo guideline development. For intra‐rater reliability assessment, 38 images were duplicated, yielding 131 images for evaluation. Single‐angle evaluation used only the 45° oblique images, whereas multi‐angle evaluation combined three images into a single image set.

### 2.3 Scale Development

A five‐grade photonumeric CSS was developed to assess cheek skin smoothness and surface texture, with each grade defined as follows: Grade 0 (none), smooth visual skin texture; Grade 1 (minimal), slightly rough and uneven visual skin texture; Grade 2 (moderate), moderately rough and uneven visual skin texture (may have early elastosis); Grade 3 (severe), severely rough visual skin texture, crosshatched fine lines (may have mild elastosis); and Grade 4 (very severe), extremely rough visual skin texture, crosshatched deep line (severe elastosis).

Although elastosis is referenced in the descriptions of selected higher grades, it was not used as a defining criterion for CSS grade assignment. Grading was based primarily on visually assessed skin surface roughness, surface uniformity, and fine line morphology. Representative clinical photographs and written descriptors for Grades 0–4 are provided to ensure consistent and reproducible assessments (Table 1).

**TABLE 1:** Grade definitions for the CSS.

Grade	Description
**0—None**	Smooth visual skin texture
**1—Minimal**	Slightly rough and uneven visual skin texture
**2—Moderate**	Moderately rough and uneven visual skin texture; may have early elastosis
**3—Severe**	Severely rough visual skin texture, crosshatched fine lines; may have mild elastosis
**4—Very severe**	Extremely rough visual skin texture, crosshatched deep line; severe elastosis

Abbreviation: CSS, Cheek Smoothness Scale.

### 2.4 Raters and the Rating Process

Five board‐certified dermatologists participated as raters. Before the reliability assessment, all raters completed standardized CSS training, including detailed definitions and representative reference images for each grade to ensure consistent interpretation of the scale.

For the single‐angle analysis, 93 photographs captured at 45° and 38 duplicates were evaluated in random order, totaling 131 images. For the multi‐angle analysis, 93 image sets (each containing 0°, 45°, and 90° views) and 38 duplicate sets were evaluated, yielding 131 sets in total.

The representative anchor images used to define CSS grades and those used for rater training were not included in the reliability and validity evaluation datasets. The reliability analyses were performed using independent photographic cases to avoid potential inflation of agreement estimates.

### 2.5 Statistical Analysis

Inter‐rater reliability was assessed using Fleiss' kappa, and intra‐rater reliability was evaluated using Cohen's kappa based on the duplicated images [15, 16]. Kappa values were interpreted according to Altman's criteria (Table 2) [17]. All statistical analyses used two‐sided tests with a significance threshold of 0.05 and were consistent with methodologies previously used to validate aesthetic assessment scales [12, 18]. Analyses were performed using SAS version 9.4 or higher (SAS Institute Inc., Cary, NC, USA).

**TABLE 2:** Altman's criteria for agreement strength.

Kappa	Strength of agreement
< 0.20	Poor
0.21–0.40	Fair
0.41–0.60	Moderate
0.61–0.80	Good
0.81–1.00	Very good

## 3 Results

### 3.1 Inter‐Rater Reliability

For the single‐angle (45°) evaluation, inter‐rater reliability was excellent, with a Fleiss' kappa of 0.9570, indicating very good agreement among the five dermatologists. Similarly, the multi‐angle evaluation showed high inter‐rater reliability, with a Fleiss' kappa of 0.9407, indicating very good agreement among the five dermatologists. All kappa values exceeded 0.6, confirming acceptable inter‐rater reliability according to Altman's interpretation of the kappa coefficient (Table 3) [17].

**TABLE 3:** Inter‐rater reliability of the CSS.

Category	Kappa	Standard Error	95% Confidence interval	
			Lower	Upper
Single‐angle evaluation	0.9570	0.0125	0.9325	0.9815
Multi‐angle evaluation	0.9407	0.0153	0.9107	0.9707

Abbreviation: CSS, Cheek Smoothness Scale.

### 3.2 Intra‐Rater Reliability

Intra‐rater reliability for the single‐angle evaluation was consistently high, with Cohen's kappa ranging from 0.9322 to 1.0000. For the multi‐angle evaluation, intra‐rater reliability remained robust, with kappa values ranging from 0.8979 to 0.9660. All intra‐rater kappa values exceeded the threshold for very good agreement, indicating excellent consistency in repeated assessments of cheek skin smoothness and surface texture by individual raters (Table 4).

**TABLE 4:** Intra‐rater reliability of the CSS.

Category	Raters	Kappa	Standard Error	95% Confidence interval	
				Lower	Upper
Single‐angle evaluation	Rater 1	0.9665	0.0332	0.9015	1.0000
	Rater 2	0.9666	0.033	0.9019	1.0000
	Rater 3	0.9332	0.0459	0.8431	1.0000
	Rater 4	0.9664	0.0330	0.9017	1.0000
	Rater 5	1.0000	0.0000	1.0000	1.0000
Multi‐angle evaluation	Rater 1	0.9659	0.0336	0.9000	1.0000
	Rater 2	0.8979	0.0568	0.7865	1.0000
	Rater 3	0.9320	0.0469	0.8401	1.0000
	Rater 4	0.9660	0.0336	0.9001	1.0000
	Rater 5	0.9660	0.0336	0.9001	1.0000

Abbreviation: CSS, Cheek Smoothness Scale.

### 3.3 Agreement Between Single‐ and Multi‐Angle Evaluations

Both single‐ and multi‐angle evaluations met the predefined criteria for good to very good agreement according to Altman's interpretation of the kappa coefficient, confirming high inter‐ and intra‐rater reliability of the CSS. Among the 93 nonduplicated cases, complete agreement between single‐ and multi‐angle assessments demonstrated a high level of concordance between the two approaches.

**FIGURE 1:**
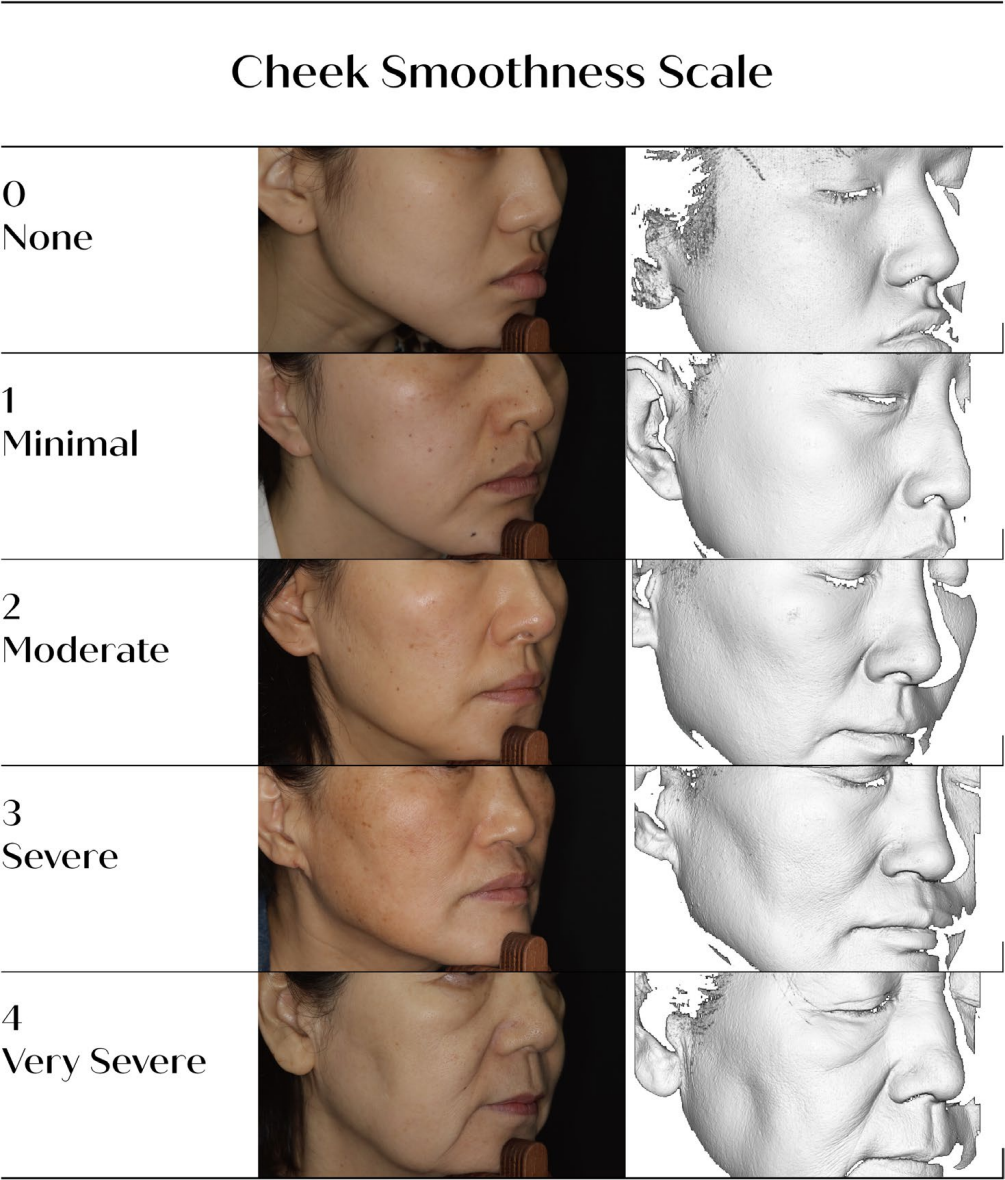
Cheek Smoothness Scale (CSS).

Based on these findings, the final photographic grades for the CSS were established (Figure 1), and the 45° oblique view was confirmed as a representative and practical angle for assessing cheek skin texture.

### 3.4 Instrument‐Based Validation Using PRIMOS Ra Values

**FIGURE 2:**
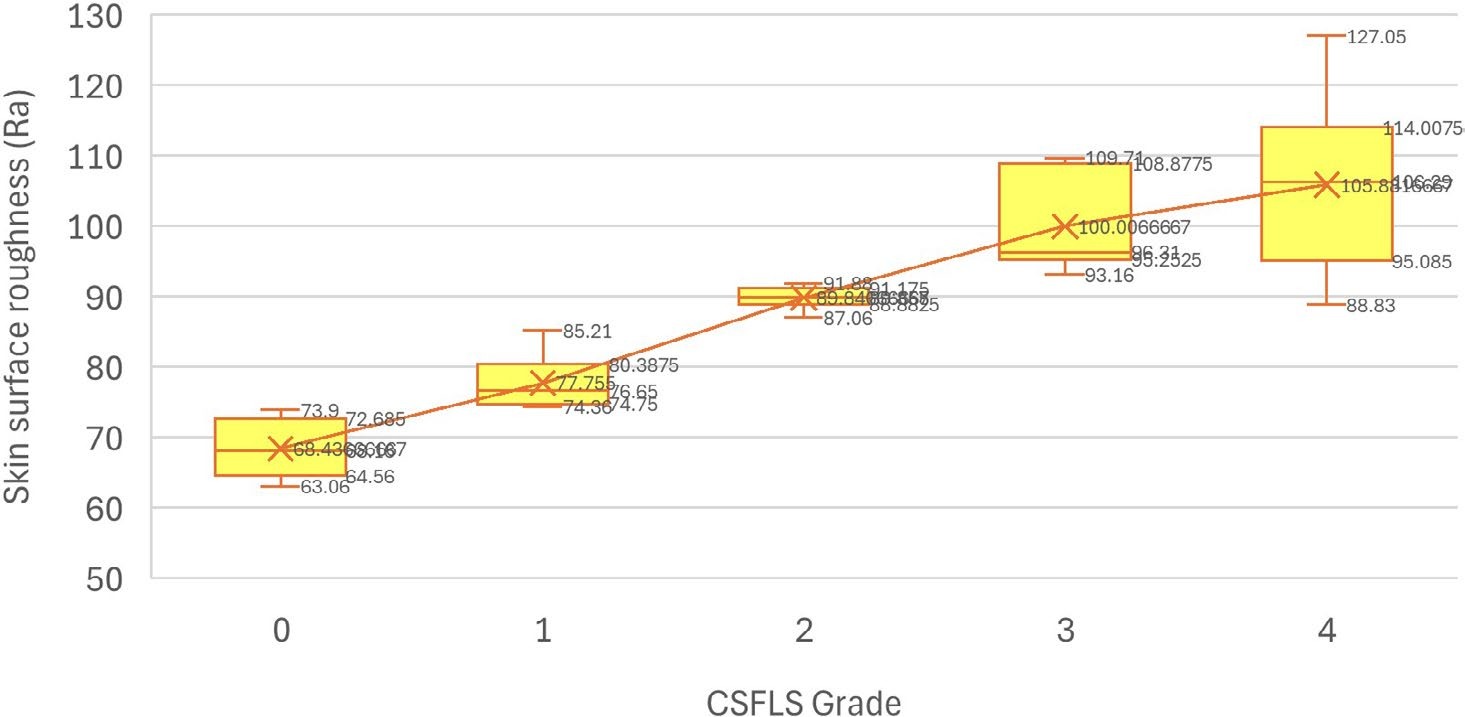
Correlation between CSS grades and PRIMOS‐derived skin surface roughness. CSS, Cheek Smoothness Scale.

As an additional objective validation, the representative clinical photographs used for scale development were analyzed using a PRIMOS CR system (GFMesstechnik GmbH, Berlin, Germany) to obtain skin surface roughness measurements expressed as Ra values. The analysis showed a progressive increase in Ra values with increasing CSS grade, indicating that higher visual grades were associated with greater skin surface roughness (Figure 2). This monotonic relationship supports the construct validity of the CSS, demonstrating a strong concordance between visually assessed cheek skin smoothness and objectively measured skin surface roughness.

**FIGURE 3:**
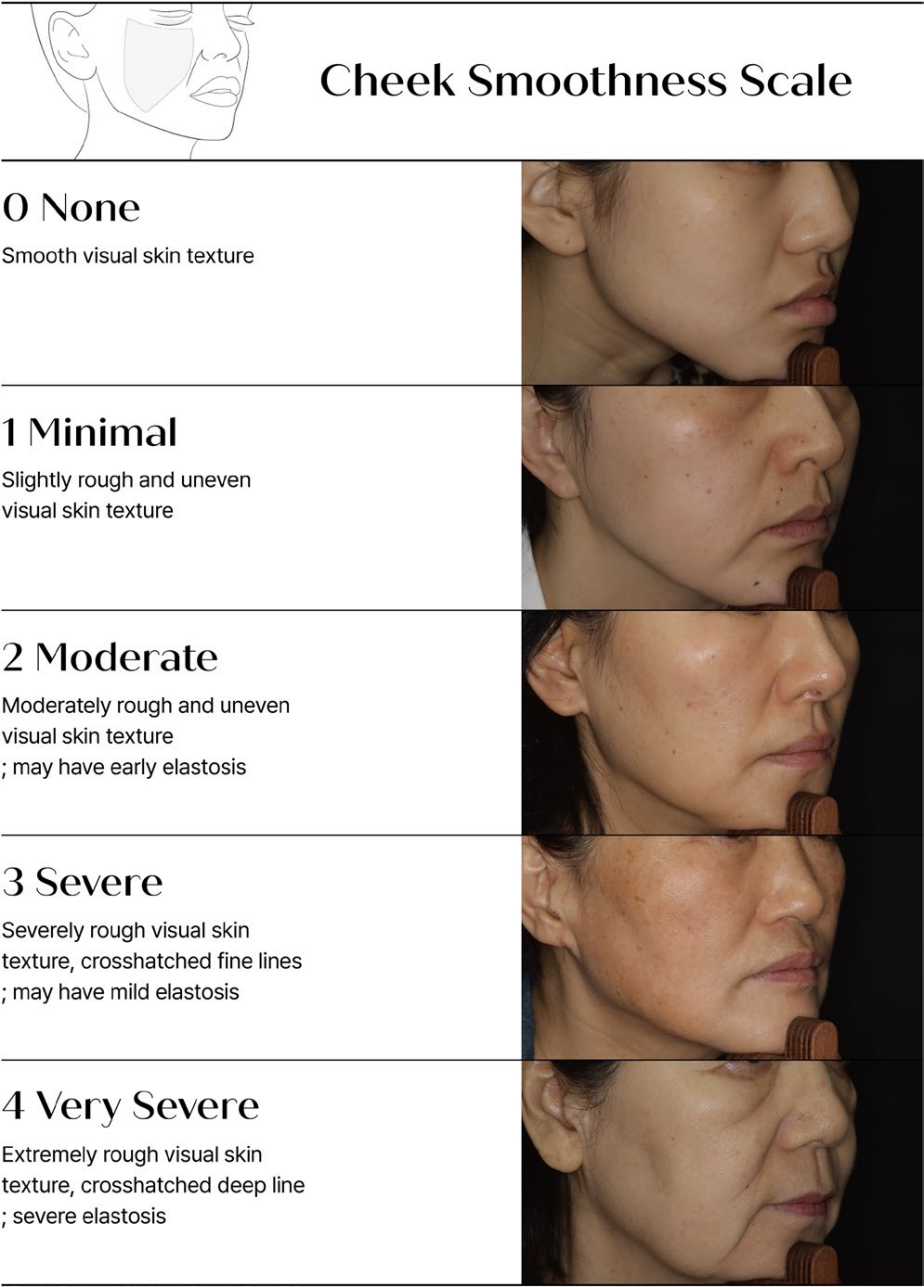
Representative clinical photographs along the corresponding PRIMOS CR images for each CSS grade. CSS, Cheek Smoothness Scale.

To further enhance clinical interpretability, PRIMOS CR images corresponding to representative photographs for each CSS grade were presented alongside conventional clinical images (Figure 3). These PRIMOS images enabled clearer visualization of subtle surface irregularities and textural differences between CSS grades, particularly between adjacent CSS categories, thereby facilitating intuitive discrimination of cheek skin texture severity. This combined presentation may serve as an auxiliary reference tool in real‐world clinical practice.

## 4 Discussion

In this study, we developed and validated the CSS, a five‐grade photonumeric scale for assessing cheek skin smoothness and surface texture using standardized clinical photography. This scale was designed to address the clinical need for a cheek‐specific, texture‐focused assessment of skin surface smoothness using standardized clinical photography. The results demonstrated excellent inter‐ and intra‐rater reliability for single‐ and multi‐angle evaluations, with kappa values consistently within the good‐to‐very good range according to Altman's criteria. These findings support the CSS as a reliable and reproducible tool for visually assessing cheek skin texture in both clinical and routine aesthetic practice.

The methodological approach in this study, based on Fleiss' kappa statistics, aligns with validation frameworks previously applied to photographic scales assessing photo damage, wrinkle severity, glabellar frown lines, and other facial aesthetic attributes [19, 20, 21, 22, 23]. Employing this well‐established statistical methodology strengthens the robustness of the reliability analysis and supports the broader applicability of the CSS in aesthetic dermatology.

Conceptually, the CSS aligns closely with photonumeric assessment frameworks for evaluating skin texture and surface roughness, most notably the Allergan Skin Roughness Scale [14]. However, while the Allergan Skin Roughness Scale demonstrates excellent reliability and clinical validity, its validation cohort is predominantly Caucasian, with Asian individuals representing only a small proportion of the study population. Facial aging patterns vary across ethnic groups. Asian skin is generally characterized by a thicker dermis, higher collagen density, delayed wrinkle formation, and greater prominence of textural roughness and fine lines rather than deep furrows [24, 25, 26]. Accordingly, further validation of skin quality assessment scales in Asian populations remains warranted.

A recent large‐scale comparative study shows that photonumeric scales validated in Caucasian populations demonstrate moderate to strong concordance with Chinese‐validated scales when applied to Asian populations across several facial regions, including crow's feet, forehead wrinkles, glabellar frown lines, and nasolabial folds [27]. These findings support the broader cross‐ethnic applicability of established frameworks for facial wrinkle assessment. However, the comparative study did not evaluate cheek skin texture or surface smoothness, which represents more diffuse and distinct aging features than those of discrete wrinkle‐prone regions. In this context, the present study did not create an ethnicity‐specific scale but rather validated a cheek‐specific photonumeric scale grounded in established skin texture assessment concepts within an Asian population. While the current validation focuses on Asian subjects, the underlying framework of the CSS is intended to be broadly applicable, and further studies are warranted to confirm its performance across diverse ethnic populations.

Beyond ethnicity‐specific considerations, earlier photonumeric scales were developed using imaging technologies that differ from contemporary high‐resolution clinical photography, which may limit their practicality and clinical interpretability in real‐world aesthetic practice. This limitation is particularly relevant for assessing cheek skin texture, where age‐related changes typically manifest as subtle, gradual alterations in surface smoothness and roughness that may be difficult to discern using lower‐resolution reference images. Therefore, incorporating additional objective, high‐resolution measures is important for reinforcing the validity of visual grading in this region.

To address this need, objective skin surface analysis using the PRIMOS CR system was incorporated. PRIMOS‐derived Ra values increased progressively with higher CSS grades, indicating close correspondence between the visual grading system and quantitative changes in skin surface roughness. These findings support the construct validity of the CSS, demonstrating that visually assessed cheek skin smoothness aligns with objective, instrument‐based measurements of skin texture. PRIMOS CR images are also presented alongside representative clinical photographs as a qualitative visual adjunct. This combined presentation enables subtle surface irregularities and fine textural differences to be more readily appreciated, particularly between adjacent grades. While PRIMOS imaging is not intended as a standalone quantitative assessment tool, its parallel use may enhance clinical interpretability and facilitate more intuitive application of the CSS in routine practice.

This study has some limitations. All assessments were conducted by board‐certified dermatologists, which may limit the generalizability of reliability to evaluators with different levels of clinical experience. Future studies involving more diverse evaluator groups and broader populations would further strengthen the external validity of the scale. In addition, the responsiveness of the CSS to longitudinal, treatment‐related changes was not evaluated. Longitudinal validation studies are therefore warranted to confirm the sensitivity of the scale to clinically meaningful improvements over time.

In conclusion, the CSS is a reliable and reproducible five‐grade photonumeric assessment tool for evaluating cheek skin smoothness and surface texture, demonstrating excellent inter‐ and intra‐rater reliability. The scale is conceptually grounded in established skin texture assessment frameworks and is further supported by objective validation using PRIMOS CR‐derived skin surface roughness measurements, confirming its construct validity. By capturing clinically relevant changes in cheek skin smoothness, the CSS may serve as a standardized outcome measure for evaluating treatment efficacy in clinical trials, device‐based intervention studies, and post‐treatment assessments in routine aesthetic practice. In addition, its validated and reproducible structure supports potential use in regulatory‐facing clinical studies requiring objective and consistent visual endpoints. Further studies involving diverse populations and longitudinal treatment assessments are warranted to expand its applicability and to confirm responsiveness to clinically meaningful changes over time.

## Author Contributions

H.S.H. and B.J.K.: conception and design; H.S.H. and B.J.K.: analysis and interpretation of the data; H.S.H.: drafting of the paper; H.S.H. and B.J.K.: revising the manuscript critically for intellectual content; all authors were involved in the final approval of the version to be published and agreed to be accountable for all aspects of the work.

## Funding

This work was funded by PharmaResearch Co. Ltd., Republic of Korea.

## Ethics Statement

The study was approved by the institutional review board (IRB No. P2504‐8565). All participants consented to the reproduction and distribution of any images collected during the study.

## Consent

Written informed consent was obtained from all individual participants included in the study.

## Conflicts of Interest

The authors declare no conflicts of interest.

## Data Availability

The data that support the findings of this study are available on request from the corresponding author. The data are not publicly available due to privacy or ethical restrictions.
